# Infectious Fevers and Diseases in London

**Published:** 1886-10-30

**Authors:** 


### Infectious Fevers and Diseases in London.

**The Last Month.** During the four weeks ending Friday, October 22nd, London has been notably free from small-pox; but four cases were admitted during this period into the hospitals of the Metropolitan Asylums Board, and they came from widely-scattered districts. As yet, there is no indication that London is to be visited during the winter with any special prevalence of this disease. Scarlet fever, on the other hand, has asserted itself in increasing proportions, and as many as 284 cases of this disease were admitted into the Asylums Board's Institutions. Of these, Hackney contributed 38, Lambeth 31, and St. Pancras 29. Not a single case of typhus fever was admitted during the same period—a result not uncommon at the present time, but one which, not many years ago, would have been learnt with surprise. The practical disappearance of this disease from London is due to the removal from its midst of the miserable dwellings which, year after year, used to serve as centres from which the malady spread. Enteric fever contributed 69 patients to the Board's hospitals; they were fairly evenly distributed over the Metropolis, and it may therefore be assumed that there is no special influence in operation, such as the pollution of a local water or milk supply. The accompanying Table gives in more detail the nature of the diseases from which the patients admitted to the hospitals suffered, and the localities from which they have come.

*Return of Cases admitted into the Hospitals of the Metropolitan Asylums Board during the four weeks ending October 22nd, 1886.*

No case of Typhus Fever was admitted to treatment.

| Sanitary District. | Name of Disease. | | |
|---|---|---|---|
| | Small-Pox. | Scarlet Fever. | Enteric Fever. |
| Bermondsey | .. | 2 | .. |
| Bethnal Green | .. | 6 | 7 |
| Camberwell | .. | 7 | 2 |
| Chelsea | .. | 2 | .. |
| City of London | .. | 1 | .. |
| Clerkenwell | .. | 7 | 1 |
| Fulham | 1 | 8 | 7 |
| Greenwich | .. | 3 | 2 |
| Hackney | .. | 38 | 11 |
| Hammersmith | .. | 6 | 1 |
| Hampstead | .. | 1 | .. |
| Holborn | .. | 4 | 1 |
| Islington | .. | 10 | 6 |
| Kensington | .. | 5 | .. |
| Lambeth | .. | 31 | 1 |
| Lewisham | .. | 1 | .. |
| Limehouse | .. | 7 | 3 |
| Mile End | .. | 6 | .. |
| Newington | 1 | 3 | 1 |
| Plumstead | .. | 3 | .. |
| Poplar | 1 | 3 | 4 |
| Rotherhithe | .. | 1 | .. |
| Shoreditch | .. | 6 | 6 |
| Southwark | .. | 4 | .. |
| Stepney | .. | 4 | 1 |
| St. George's, Bloomsbury | .. | 3 | 1 |
| " Hanover Sq. | .. | 12 | .. |
| " in-the-East | .. | 7 | 1 |
| St. James, Westminster | .. | 3 | .. |
| St. Luke's | .. | 11 | 2 |
| St. Martin's-in-the-Fields | .. | 1 | .. |
| St. Marylebone | .. | 12 | 2 |
| St. Olave's | .. | 1 | .. |
| St. Pancras | .. | 29 | 1 |
| St. Saviour's | .. | 2 | 1 |
| Wandsworth | 1 | 14 | 4 |
| Westminster | .. | 12 | .. |
| Whitechapel | .. | 8 | 3 |
| Totals | 4 | 284 | 69 |

**Diphtheria or Fever.** An interesting point has just been raised between the Metropolitan Asylums Board and the Local Government Board. The former Board has been requested to receive patients suffering from diphtheria, and appears willing to undertake this duty, but the important question had to be settled as to whether diphtheria could be included as a "fever" within the meaning of section 69 of the Metropolitan Poor Act of 1867. The managers appealed to the Local Government Board, who, in their turn, sought the opinion on this point of the College of Physicians; and the President of the College, Sir William Jenner, in reply, stated that "if the words in the Act had been for persons suffering from 'fevers,' I should most certainly have considered diphtheria to be included under the term 'fever'; but the separation of small-pox from fevers seems to signify that the word 'fever' was intended to include only fever of a special type, those cases, that is to say, to which the word 'fever' is specially applied, as scarlet fever, typhus fever, and typhoid fever." The Local Government Board have now decided that there must be special legislation for the purpose of admitting cases of diphtheria into the hospitals of the managers, and they will consider whether such legislation should be proposed.

**Fever Hospitals for all Classes.** This question serves well to exemplify the fact that the hospitals of the managers have only come by force of circumstances to be regarded as a sanitary defence of the Metropolis. The whole intention of the legislature was to provide accommodation for persons who were paupers, and the effect of this accommodation, whether for good or evil, upon the public health was not considered. In seeking to receive cases of diphtheria, the managers are taking a step which distinctly indicates their desire to extend their usefulness to the limitation of all dangerous infectious maladies. In striking contrast to this action is that of the Local Sanitary Authorities who have been invited to contract with the managers for the reception into their hospitals of persons who are not paupers; but fourteen of these Authorities have accepted this invitation, fifteen have refused, seven have returned evasive answers, and four have given no replies. At present large numbers of persons not paupers are sent to the hospitals on the orders of relieving officers. When thus admitted, only the cost of their maintenance falls upon the parish which sends them, the other expenses falling upon a common Metropolitan fund. In contracting with the managers, each parish would probably become liable for a larger share of the total money expended upon every patient it sends, than if the patient were admitted as a pauper on the relieving officer's order. But another argument has probably greater weight than this with some parochial authorities: no satisfactory definition of a pauper can be formulated, and if the question arose for each individual patient whether he should be sent to hospital through the poor-law or sanitary officials, serious delay might occur while this point was being decided. The difficulty, no doubt, lies in the fact that these hospitals were originally intended for paupers only; but the whole subject is of so much importance that we propose to refer to it again, and we therefore need not discuss it further in the present number.

